# Combined reflectance spectroscopy and stochastic modeling approach for noninvasive hemoglobin determination via palpebral conjunctiva

**DOI:** 10.1002/phy2.192

**Published:** 2014-01-08

**Authors:** Oleg Kim, John McMurdy, Gregory Jay, Collin Lines, Gregory Crawford, Mark Alber

**Affiliations:** 1 Department of Applied and Computational Mathematics and Statistics, University of Notre Dame, Notre Dame, 46556, Indiana; 2 Division of Engineering, Brown University, Providence, 02912, Rhode Island; 3 Department of Emergency Medicine and Division of Engineering, Brown University, Providence, 02912, Rhode Island; 4 Department of Physics, University of Notre Dame, Notre Dame, 46556, Indiana; 5 Department of Medicine, Indiana University School of Medicince, Indianapolis, 46202, Indiana

**Keywords:** Blood, hemoglobin, modeling, reflectance spectroscopy

## Abstract

A combination of stochastic photon propagation model in a multilayered human eyelid tissue and reflectance spectroscopy was used to study palpebral conjunctiva spectral reflectance for hemoglobin (Hgb) determination. The developed model is the first biologically relevant model of eyelid tissue, which was shown to provide very good approximation to the measured spectra. Tissue optical parameters were defined using previous histological and microscopy studies of a human eyelid. After calibration of the model parameters the responses of reflectance spectra to Hgb level and blood oxygenation variations were calculated. The stimulated reflectance spectra in adults with normal and low Hgb levels agreed well with experimental data for Hgb concentrations from 8.1 to 16.7 g/dL. The extracted Hgb levels were compared with in vitro Hgb measurements. The root mean square error of cross‐validation was 1.64 g/dL. The method was shown to provide 86% sensitivity estimates for clinically diagnosed anemia cases. A combination of the model with spectroscopy measurements provides a new tool for noninvasive study of human conjunctiva to aid in diagnosing blood disorders such as anemia.

## Abstract (precis)

Our study describes a novel method combining stochastic modeling and experimental spectroscopy to address an important biomedical problem of noninvasive hemoglobin assessment via palpebral conjunctiva. The developed model is the first biologically relevant detailed representation of light propagation through eyelid tissue, which was shown to provide very good predictions for the measured spectra.

## Introduction

Anemia is a condition in which the number of red blood cells or the amount of hemoglobin (Hgb) is below normal. According to World Health Organization, persons with concentrations less than 13 g/dL for men and 12 g/dL for women are defined to be anemic. Anemia affects almost 3.5 million Americans, with millions more being undiagnosed, making it the most common blood disorder in the United States (Goodnough and Nissenson 2004). Anemia can be induced by malaria, a mosquito‐borne infectious disease caused by the Plasmodium parasite. Each year severe malarial anemia causes between 190,000 and 974,000 deaths among children less than 5 years (Murphy and Breman 2001) of age. Anemia is also the most common complication seen in 80% people with AIDS/HIV (Volberding et al. 2004).

There exist different types of anemia ranging from mild and easily treatable iron and vitamin deficiency anemias to serious life‐threatening aplastic and sickle cell anemias. The main anemia symptoms affecting physical function are fatigue, weakness, and fainting. Meanwhile, mild anemia may occur without symptoms and can be detected only during a medical exam including a blood test.

Current clinical methods to diagnose anemia rely on invasive determination of blood Hgb. Generally, the complete blood count (CBC) test is performed, which requires a venipuncture and a hematological analysis of the collected blood. Although the CBC test is very accurate, it has certain weaknesses such as a high cost of labor and instrumentation, and time‐expensive analysis of the test, which frequently eliminates it from routine physical exams. In addition, because of the invasiveness of the test, the phlebotomist is exposed to risk of interacting with blood‐born diseases. It is clear that a noninvasive method allowing fast and easy determination of Hgb is needed.

There exist a number of noninvasive optical methods to indirectly measure the amount of Hgb and blood oxygenation levels in human tissue. These include retinal imaging (Rice et al. 2002), blood oxygenation monitoring using continuous NIR spectrometry (Liu et al. 1999); Benni 2002), photoplethysmography (Aldrich et al. 2002), ultraviolet and visible spectroscopies (Mourant et al. 2005); Bigio and Mourant 1999), as well as fluorescence spectroscopy of oral tissue (Pavlova et al. 2009). (A detailed review of different noninvasive methods of total Hgb determination is presented in [McMurdy et al. 2008)]). Tissue reflectance spectroscopy allows one to obtain optical properties of the tissue by measuring its reflectance spectra. Reflectance spectroscopy was previously implemented to characterize cutaneous tissues (Bigio et al. 2000); Mourant et al. 2005); Randeberg et al. 2010), 2011); Kim et al. 2012); Klein et al. 2012), which are easily accessible, but present difficulties when monitoring Hgb signal due to interracial/interpatient melanin fluctuations, high scattering in turbid media, and weak signals due to skin tissue absorption at visible and near infra‐red wavelengths. These factors can potentially influence the necessary instrumentation and inhibit the acceptance of noninvasive technology by clinicians.

Human palpebral conjunctiva represents an easily accessible site for noninvasive Hgb level monitoring due to proximity of blood capillaries to an eyelid internal surface, which does not contain strong light absorbing chromophores such as melanin.

There have been several studies conducted to evaluate Hgb level from conjunctiva spectra (Ernsting et al. 2001); McMurdy et al. 2006); Suner et al. 2007). The feasibility of applying image analysis algorithms to digital photographs of the conjunctiva to predict Hgb concentrations was examined with moderately successful results (Ernsting et al. 2001). Conjunctival reflectance spectra coupled with a partial least‐squares (PLS) analysis and a discrete spectral region model were used (McMurdy et al. 2006) to derive Hgb concentration and compared to in vitro measurements. Although these studies provided some correlation between Hgb concentration and reflectance, they did not use a physiologically based model to reproduce the spectra of conjunctiva, and thus miss information about conjunctiva structural features which is important for spectral analysis when assessing Hgb levels.

Because the tissue structure is complex, determining chromophore concentrations from the spectra involves the solution of an inverse problem, which is a nontrivial task (Pavlova et al. 2009); Phelps et al. 2010). The goal of this study was to describe a novel method combining reflectance spectroscopy and a stochastic modeling approach of light propagation in multilayered tissue (Wang et al. 1995); Churmakov et al. 2002), 2003); Meglinski and Matcher 2002), 2003); Kim et al. 2012) to determine the reflectance of the palpebral conjunctiva. This will aid in evaluating the concentration of blood Hgb level and thus, in making a correct patient‐specific medical diagnosis.

Common approaches to model light propagation in biological tissues are based on the diffusion approximation (DA) of a radiative transport equation and the stochastic Monte Carlo (MC) method. In comparison to MC method, DA is computationally less expensive and can be implemented for complex geometries. However, DA underestimates the light energy deposition in the epidermis and vessels when compared with MC (Zhang et al., 2005b). The difference is nonlinearly dependent on the wavelength, dermal blood volume fraction, vessel size, and depth. It was also shown (Flock et al. 1989) that the predictions made using DA became increasingly inaccurate as the albedo tended to zero and as the scattering anisotropy factor became large (*g* *≥* 0.1) (Tuchin 2007), which is typical for biological tissues where *g*∼0.6−0.9. Meanwhile, MC method does not have these limitations. Another chief advantage of MC approach is that complex geometries and inhomogeneities can be modeled in a straight forward manner, and that a variety of physical quantities can be scored during the same run.

The novelty of our extension of the MC‐based modeling approach used in this study lies in its modified representation of the conjunctiva physiology (including spectral characteristics of conjunctival epithelium). Our novel solution method of the inverse problem quantifies Hgb level by minimizing the reflectance error over the broad wavelength range from 400 to 660 nm, whereas in previous models only several wavelengths were considered (Phelps et al. 2010). To study interaction of light photons with an eyelid, we developed an eye tissue model consisting of seven layers. In this model, each layer is characterized by a number of different parameters including chromophore concentrations, layer thickness, refraction and anisotropy indices, and scattering and absorption coefficients. A stochastic Monte Carlo‐based method is used to simulate light propagation through the tissue and conjunctival reflectance spectra are calculated and compared with experimental data. By fitting the measured and calculated reflectance curves, blood Hgb level is determined.

The rest of the study is organized as follows. Materials and Methods section describes experimental procedure of human conjunctiva reflectance spectroscopy. Then, the Tarsal Conjunctiva Model provides details of the seven‐layer tissue model. Next, the MC Method of photon propagation is described. The Results and Discussion section describes model simulation results, comparison between the simulated and experimentally measured spectra for different Hgb levels and discusses limitations and further extension of the approach. Finally, conclusions about the promising performance of the suggested noninvasive method and anemia sensitivity screening are made. This justifies the practical application of the approach that can potentially result in a valuable diagnostic tool for medicine.

## Material and Methods

**Figure 1.**
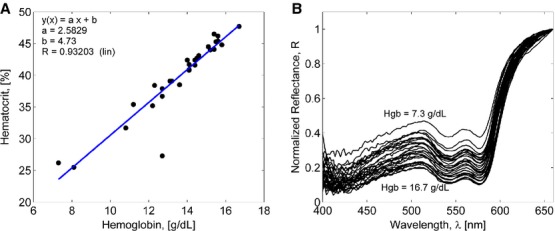
(A) The relationship between measured hematocrit and hemoglobin values. Abscissa shows hemoglobin values in grams per decaliter and ordinate shows the corresponding hematocrit values in terms of the volume percentage. Symbols are data and the line is a linear regression. (B) Experimental reflectance spectra measured by a grating spectrometer in different patients with hemoglobin concentration between 7.3 and 16.7 g/dL.

To calibrate the model, we used the spectral data collected from prior patient data. Patients enrollment and collection of conjunctiva spectra are described in detail in our previous work (McMurdy et al. 2006). Briefly, 32 patients were enrolled at the Emergency Department at Rhode Island Hospital Providence, Rhode Island. Patients with a variety of complaints were enrolled to simulate a random patient population. Patients with acute cardiac symptoms, musculoskeletal injuries inhibiting comfortable relocation to the research location, symptoms of stroke, patients not yet seen or cleared by a physician, and critically ill patients were excluded from this study. Of the 32 consented patients, 30 were able to participate (14 male and 16 female). Two patients were unable to participate; one due to nausea and abdominal pain during the enrollment procedure, and one because the conjunctiva could not be sufficiently exposed for spectroscopic measurements. Twenty‐one of these patients were Caucasian, six were Hispanic, and three were African‐American. Additionally, only patients with a minimum oxygen saturation of 90% were enrolled to exclude hypoxic patients during preliminary evaluation of the efficacy of this technique. The mean oxygen saturation was 98.3 ± 1.5%. From CBC tests of 30 patients, the mean Hgb concentration was determined to be 13.9 ± 2.1 g/dL and the mean hematocrit was 40 ± 6.0. The correlation between Hgb and hematocrit values is shown in Figure 1A which is used as an input parameter for the model. Patient's conjunctiva was illuminated at a separation of ~15 cm from source element to conjunctiva. A quartz‐tungsten‐halogen source (Lot‐Oriel, Surrey, UK) with total broadband power of 30 W was used for all measurements. The power level was chosen such that the procedure would not cause discomfort to a patient. The irradiance of this source did not exceed 0.1 W/(nm m^2^) over the visible wavelength range. According to ANSI Z136.1 standards for incoherent radiation exposure, the procedure did not pose a radiation exposure risk (Charschan and Rockwell 1999). All reflectance spectra (Fig. 1B) were collected with an aperture size of 1‐deg field of view (an aperture size of ~4.5 mm^2^). The separation distance from spectrometer to conjunctiva was 7 cm. Although a smaller aperture would be more suited to collect reflectance spectra from single capillary vessels, the random movements of patients during acquisition did not permit this procedure.

### Tarsal conjunctiva model

#### Physiology of tarsal conjunctiva

The conjunctiva is a mucosal membrane lining the eyelid (palpebral conjunctiva), the fold in the eyelid (forniceal), and the sclera (bulbar conjunctiva). Palpebral conjunctiva is an attractive location to diagnose anemia as it represents a highly vascular area with a number of surface capillaries and the mucous membrane of the conjunctiva is highly transparent. Physiologically, palpebral conjunctiva is subdivided into margin conjunctiva, tarsal conjunctiva, and orbital conjunctiva. Tarsal conjunctiva overlying the internal surface of the tarsal plate is the best location for determining Hgb through spectroscopy measurements. This region has a thin epithelium layer containing no melanin, which does not interfere the extraction of Hgb signal from reflectance spectra.

**Figure 2.**
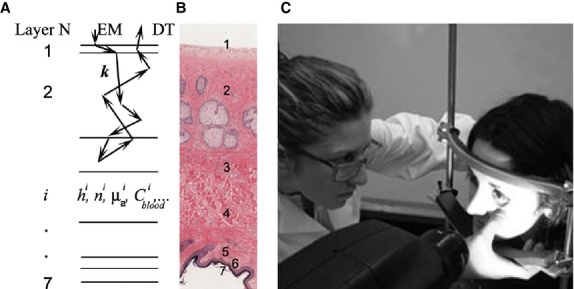
(A) A multilayered tissue model approximates a lower eyelid with seven layers which optical properties are specified in Table 1. Photons generated by a light emitter (EM) propagate in a tissue and the reflectance signal is measured by a spectroscope detector (DT). (B) Lower eyelid histology (http://histology.med.umich.edu/medical/eye) showing layers of conjunctival epithelium (1), tarsal plate (2), orbicularis oculi (3), subcutaneous fat (4), dermis (5), living epidermis (6), and stratum corneum (7). (C) The photograph of the data collection procedure using grating spectrometer.

Figure 2 shows the histological image of a human low eyelid at tarsal plate location. It can be subdivided into several regions: conjunctival epithelium, tarsal plate, orbicularis oculi, subcutaneous tissue, dermis, epidermis, and stratum corneum on the outside of the eyelid tissue. Tarsal conjunctiva epithelium layer contains 2–3 layers of cylindrical cells and cuboidal cells. Tarsal plate contains collagen type I with rows of fibroblasts, which are fiber‐forming cells in between collagen fibers. The orbicularis oculi is composed of striated muscle cells. It is responsible for blinking and squeezing eyelids shut. The eyelid skin is the thinnest in the body. It is represented by fat tissue, dermis, epidermis, and stratum corneum. Fat tissue consists of adipocytes, nerves, and blood vessels. Dermis mostly consists of supporting matrix which attract and retain water. Other components also include elastic and collagen fibers, blood vessels, hair follicles, sweat glands, macrophages, nerve endings, and lymphatic elements. The epidermis is comprised of several layers of keratinocytes with a superficial stratum corneum layer, which help to keep the skin hydrated by preventing evaporation of water. Conjunctiva derives its blood supply from the ascending branch of the posterior conjunctival artery. The major blood vessels of palpebral conjunctiva are located in the junction of tarsal conjunctiva and orbicularis oculi. There are also arterioles and capillary networks penetrating orbicularis oculi, tarsal plate, subcutaneous tissue, and dermis.

### Parametrization of tarsal conjunctiva

The structural complexity of a heterogeneous tissue is a factor affecting light propagation (Jacques 1996); Verkruysse et al. 1999); Meglinski and Matcher 2002); Tuchin 2007). The spatial distribution of tarsal conjunctiva chromophore components including blood is not uniform (Ryan 1983); Renkin and Michel 1984). However, one can distinguish layers in which chromophore content is roughly constant. Therefore, as a first‐order approximation, tarsal conjunctiva can be represented as a multilayered structure, with tissue components uniformly distributed within each layer. Some variability in thickness is expected from region to region of the conjunctiva and between individuals, and histological evidence in anecdotal human studies suggests this can be of order 30–70%. Nevertheless, for our study, we used these values for layer thickness to be typical for adults.

We used a seven layers tissue model to simulate light propagation through palpebral tarsal conjunctiva with the model parameters given in Table 1. The absorption coefficients for each layer takes into account the distribution of blood vessels, water and melanin and can be defined as (Meglinski and Matcher 2002)
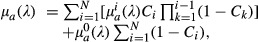
where 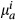 and *C*_*i*_ are the absorption coefficient and volume fraction of the *i*th chromophore, *N* is the total number of chromophores in the layer, and 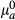 is the absorption coefficient of Hgb‐ and water‐free tissue.

**Table 1.** Optical and physiological parameters used for the tarsal conjunctiva model (Meglinski and Matcher 2002); Kakizaki et al. 2009); Cho et al. 2011); Kim et al. 2012).

Layer	Thickness, *h μ*m	*g*	*n*	*μ*_*s*_	*C*_mel_	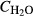	*C*_blood_	*S*	Ht, equation 7
CE	20–70	0.90	1.34	Equation 3	–	–	–	–	–
TP	100–1500	0.90	1.4	5	0	0.3	0.3–0.6	0.20–0.99	0.2–0.5
OO	2000–2500	0.95	1.4	5	0	0.3	0.3–0.6	0.20–0.99	0.2–0.5
ST	1000–1500	0.75	1.44	5	0	0.7	0.06	0.20–0.99	0.2–0.5
D	30–40	0.80	1.38	35	0	0.6	0.3	0.20–0.99	0.2–0.5
LE	10–20	0.80	1.4	45	0.01–0.1	0.2	0	0.20–0.99	0.2–0.5
SC	5–7	0.86	1.5	100	0	0.05	0	–	–

Here, *C*_mel_, 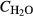 and *C*_blood_ are the volume concentrations of melanin, water and blood. *S* is oxygen saturation parameter, and Ht is hematocrit. CE, conjunctival epithelium; TP, tarsal plate; OO, orbicularis oculi; ST, subcutaneous tissue; D, dermis; LE, living epidermis; SC, stratum corneum.

Experimentally measured absorption and scattering coefficients of the conjunctival epithelium (Nemati et al. 1997), 1996) are approximated by least‐squares polynomial fits as
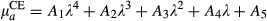
where *A*_1_ = 5.76328 × 10^−13^*A*_2_ = −5.05681 × 10^−9^, *A*_3_ = 1.09816 × 10^−5^ *A*_4_ = −0.00905007, *A*_5_ = 2.607, and
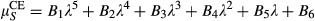
where *B*_1_ = 2.63396 × 10^−13^, *B*_2_ = −2.81535 × 10^−10^, *B*_3_ = −1.10282 × 10^−6^, *B*_4_ = 0.00236644, *B*_5_ = −1.62947, and *B*_6_ = 392.569. Here, the absorption and scattering fit errors, *R*^2^, are 0.98 and 0.9, respectively. The units of 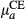 and 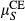 are inverse millimeters and *λ* is in nm. Polynomial coefficients *A*_*k*_ and *B*_*k*_ are calculated by minimizing the error, *R*^2^, of a polynomial fit, 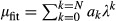. To find *a*_*k*_, the equation for a fit in matrix notation, *M*_fit_ = Λ***a***, is solved for a given set of *n* experimental data points (*λ*_*i*_, *μ*_*i*_) and the solution vector ***a*** is calculated as, ***a*** = (Λ^*T*^ Λ )^−1^ Λ^*T*^ *M*_fit_ (Nemati et al. 1997), 1996).

Absorption spectra of Hgb, water and melanin are taken from the literature (Hale and Querry 1973); Wang et al. 1995); Prahl 1999). Absorption coefficient of Hgb 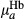 (1/cm) is calculated as follows,
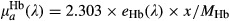
where *e* [L/(cm × mol)] is a Hgb molar extinction coefficient (Prahl et al. 1989), *x* [g/L] is the number of Hb grams per liter, and *M*_Hb_ = 64,500 (g/mol) is the Hgb gram molecular weight. The melanin absorption coefficient is given by (Jacques and McAuliffe 1991),
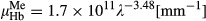
and the absorption coefficient of the Hgb‐ and water‐free tissue is approximated by (Jacques 1996),
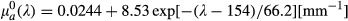
with *λ* given in nm. For blood containing layers, equation 1 yields
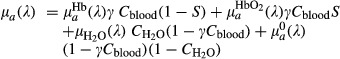
where *γ* is the fraction of Hgb in blood, *C*_blood_ is the blood volume fraction, and *S* is the oxygenation parameter. Assuming that Hgb is contained in erythrocytes only, *γ *= φ_Hb_ Ht, where φ_Hb_ is the volume fraction of Hgb in a single erythrocytes and Ht is the hematocrit level. To determine the hematocrit‐Hgb relation, the linear regression analysis based on 30 patients' CBC tests was used, which yielded the following Ht(Hgb) dependence, (see Fig. 1A) 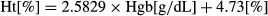


Thus, equation 6 combined with equations 2–5,5, and 7 expresses the absorption of the blood containing layers as a function of wave length, *λ*, Hgb level, Hgb, oxygenation, *S*, and known absorption coefficients 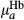, 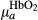, 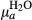 and 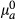.

## MC Method

**Figure 3.**
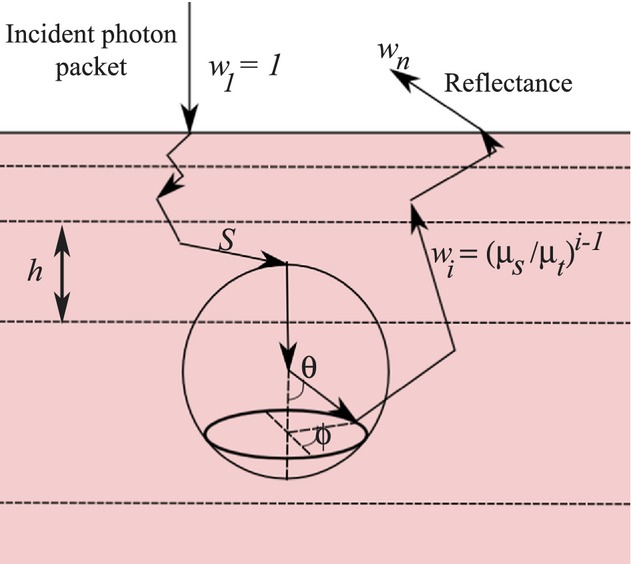
Schematic diagram of photon propagation in a multilayered model tissue.

The MC model employed the tracking of photon packets in a multilayer tissue mimicking human palpebral conjunctiva. The detailed description of the MC algorithm can be found in (Kim et al. 2012), (Wang et al. 1995). Briefly, the photon packet starts at a skin surface where radiation enters the medium and terminates when the packet is absorbed or leaves the tissue (Fig. 3). Inside the tissue the photon propagates in a random manner changing its direction of motion each time it is scattered or internally reflected. The mean free path of the photon is calculated as *S *=**−ln*ξ*_1_/(*μ*_*t*_), where *ξ*_1_ is a uniformly distributed random number from 0 to 1, *μ*_*t*_ = *μ*_*s*_ + *μ*_*a*_, with *μ*_*s*_ and *μ*_*a*_ being the scattering and absorption coefficients of the medium. At time zero each photon is assigned a unit weight. At each propagation time step, *i*, this weight is attenuated according to local absorption and scattering properties of the layer, such that a new weight is given by *w*_*i*_ = *w*_*i*‐1_ *μ*_*s*_/*μ*_*t*_. If *w*_*i*_ becomes smaller than a prescribed threshold value *w*_th_, then the photon is terminated and the next photon packet enters the tissue medium. If *w*_*i*_ is still larger than *w*_th_, a new movement direction of the photon is calculated with azimuthal and longitudinal angles calculated as *φ* = 2*πξ*_2_, and 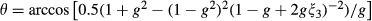 (Henyey and Greenstein 1941), where *g* is the anisotropy factor, and *ξ*_3_ is a random number uniformly distributed from 0 to 1. A photon packet directional vector is calculated according to (Prahl et al. 1989).

By accumulating the total photon weight at a detector location, the reflectance at a given wavelength is calculated as a ratio of the total reflected photon weight to a total weight of incident photons. By repeating the process for different wavelengths, the reflectance spectra is numerically obtained. In this study, the reflectance spectra from 400 to 700 nm were simulated at intervals of 2 nm. To obtain the stable solution, 10^5^–10^6^ iterations were used. Input model parameters determining the optical properties of each layer for a given wavelength included: layer thickness, absorption and scattering coefficients (*μ*_*a*_ and *μ*_*s*_), oxygenation parameter (*S*), refractive index (*n*), and anisotropy scattering parameter (*g*). *g*(*λ*) ranges from −1 to 1 corresponding to perfect backscattering and forward scattering. It is defined as an averaged cosine value of the phase function, which gives the intensity distribution of scattered light with respect to the deflection angle *θ*.

### Calibration and predictive simulations

**Figure 4.**
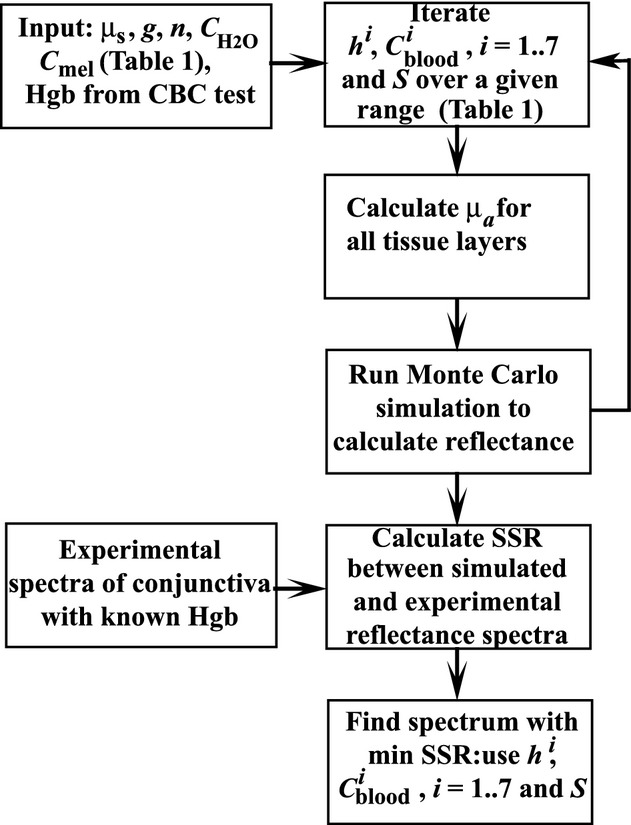
A flowchart of the main steps of model calibration: (1) the input parameters including measured Hgb level and scattering coefficients are specified for each layer, (2) the iterative procedure begins with specifying blood concentration and oxygenation levels and layer thicknesses, (3) absorption properties are calculated according to equations 1–5,1–7, (4) Monte Carlo simulations are run for specified parameters, (4) simulated and experimental spectra are compared and the sum of squared residuals (SSR) is calculated for each set of parameters, (5) minimum of SSR is found and the corresponding 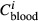, *h*_*i*_, and *S* are determined.

**Figure 5.**
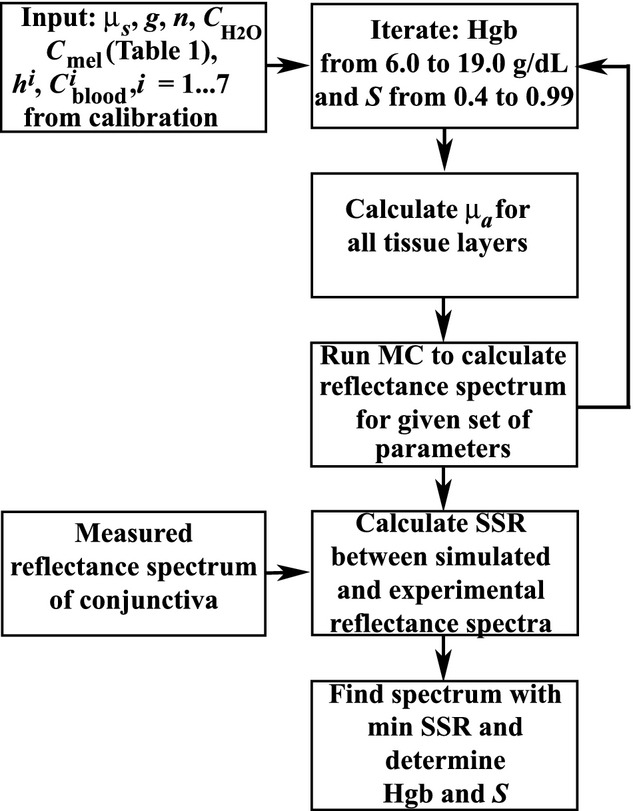
A flowchart of the main steps of predictive modeling: (1) the input parameters including those obtained from calibration are specified for each layer, (2) the iterative procedure begins with specifying Hgb level and oxygenation levels, (3) absorption properties are calculated according to equations 1–5,1–7, (4) Monte Carlo simulations are run for specified parameters, (4) simulated and experimental spectra are compared and the sum of squared residuals (SSR) is calculated for each set of parameters, (5) the minimum of SSR is found and the corresponding Hgb and *S* are determined.

The combined approach consists of two parts: model calibration (Fig. 4) and predictive simulations (Fig. 5). During calibration, first, the input parameters including the Hgb level (measured using CBC) and scattering coefficients are specified for each layer. Next, the iterative procedure starts. Each step of the iteration consists of (1) changing simulation blood concentration and oxygenation levels and thicknesses of tissue layers; (2) calculating absorption properties according to equations 1–5,1–7; and (3) running forward MC simulations for the specified layer scattering and absorption parameters. After that, simulated and experimental spectra are compared and the sum of squared residuals (SSR) is calculated for each set of parameters. Finally the minimum of SSR is found and the corresponding 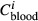, *h*_*i*_, and *S* are determined, where *h*_*i*_ is the thickness of the *i*‐th layer.

Predictive modeling is based on the iterative solution of an inverse problem using forward MC simulations. By using the calibrated input parameters and by running stochastic simulations over a specific range of Hgb and oxygenation values, the corresponding Hgb and *S* values are derived by fitting a simulated spectrum to a measured one. To find the best‐fit spectrum, the minimum of SSR is calculated for each set of parameter values.

To estimate the performance of a predictive model combined with spectroscopic measurements, correlation between predicted and measured with CBC Hgb levels is assessed. Cross‐validation is performed using the leave‐one‐out method (Boser et al. 1992). Each round of cross‐validation uses 29 spectra as a training set to predict a Hgb concentration corresponding to a single spectrum. To reduce variability, multiple rounds of cross‐validation are performed for 30 spectra, and the validation results are averaged over the rounds.

## Results and Discussion

### Sensitivity analysis

**Figure 6.**
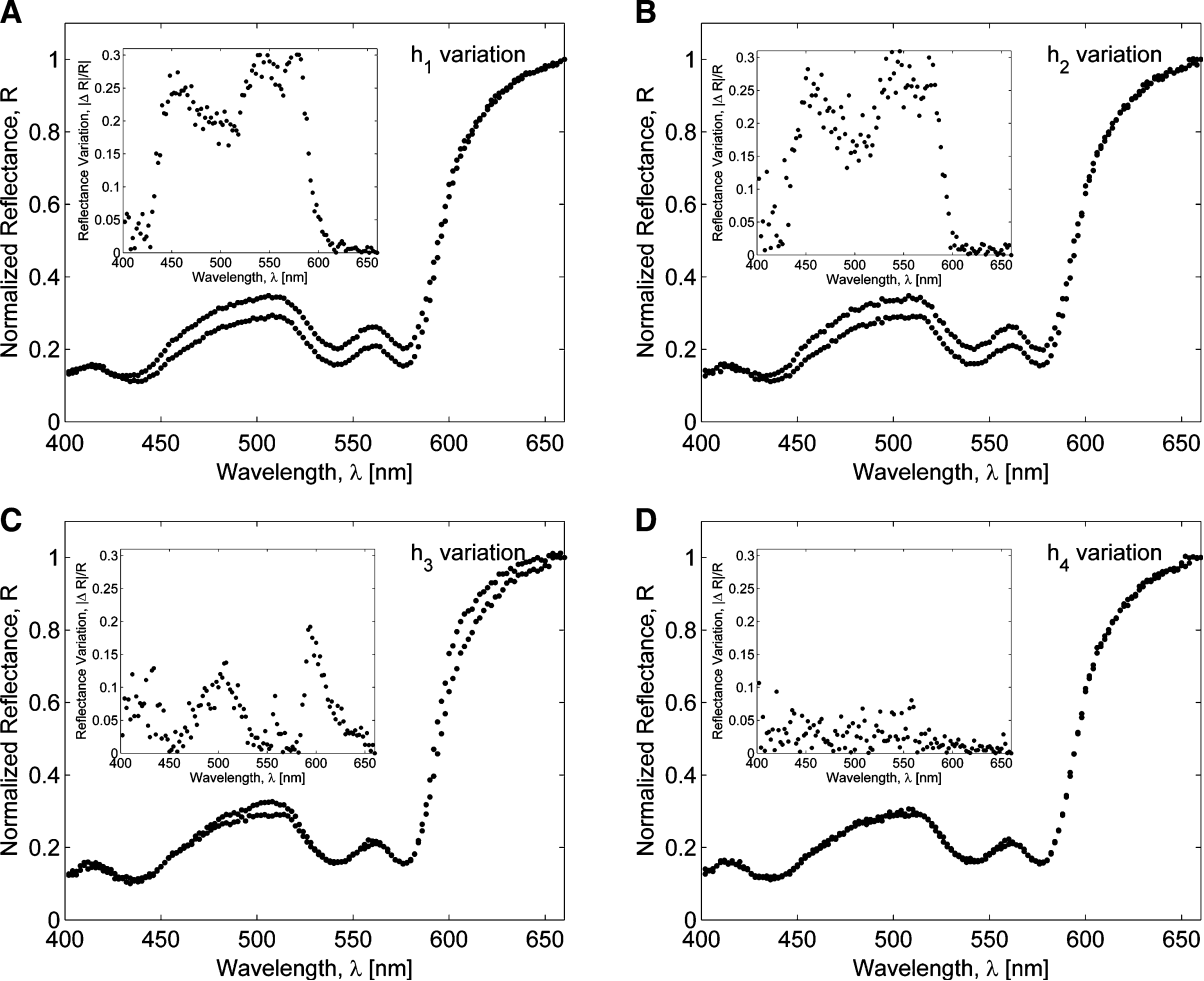
Palpebral conjunctiva reflectance spectra obtained with Monte Carlo simulations of photons transport in the tissue for various thicknesses of (A) conjunctival epithelium, *h*_1_**=**20–70 *μ*m, (B) tarsal plate *h*_2_**=**100–1000 *μ*m, (C) orbicularis oculi *h*_3_ = 2000*–*2500 *μ*m, (D) subcutaneous fat *h*_4_ = 250*–*1500 *μ*m. 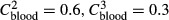, *S* = 0.9, Hgb = 15.4 g/dL. The relative variation is shown in the inset figure.

**Figure 7.**
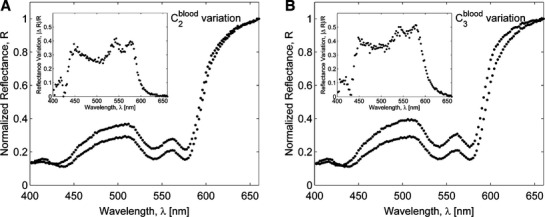
Variations in Monte Carlo simulated palpebral conjunctiva reflectance spectra due to changes in blood volume fraction in (A) tarsal plate layer *C*_blood_ = (0.08 − 0.3); (B) orbicularis oculi *C*_blood_ = (0.15 − 0.6). The relative variation is shown in the inset figure.

**Figure 8.**
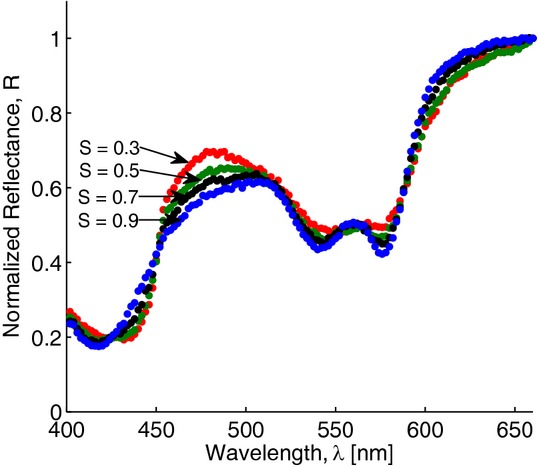
Monte Carlo simulations of palpebral conjunctiva reflectance spectra for different values of the oxygenation parameter, *S*.

**Figure 9.**
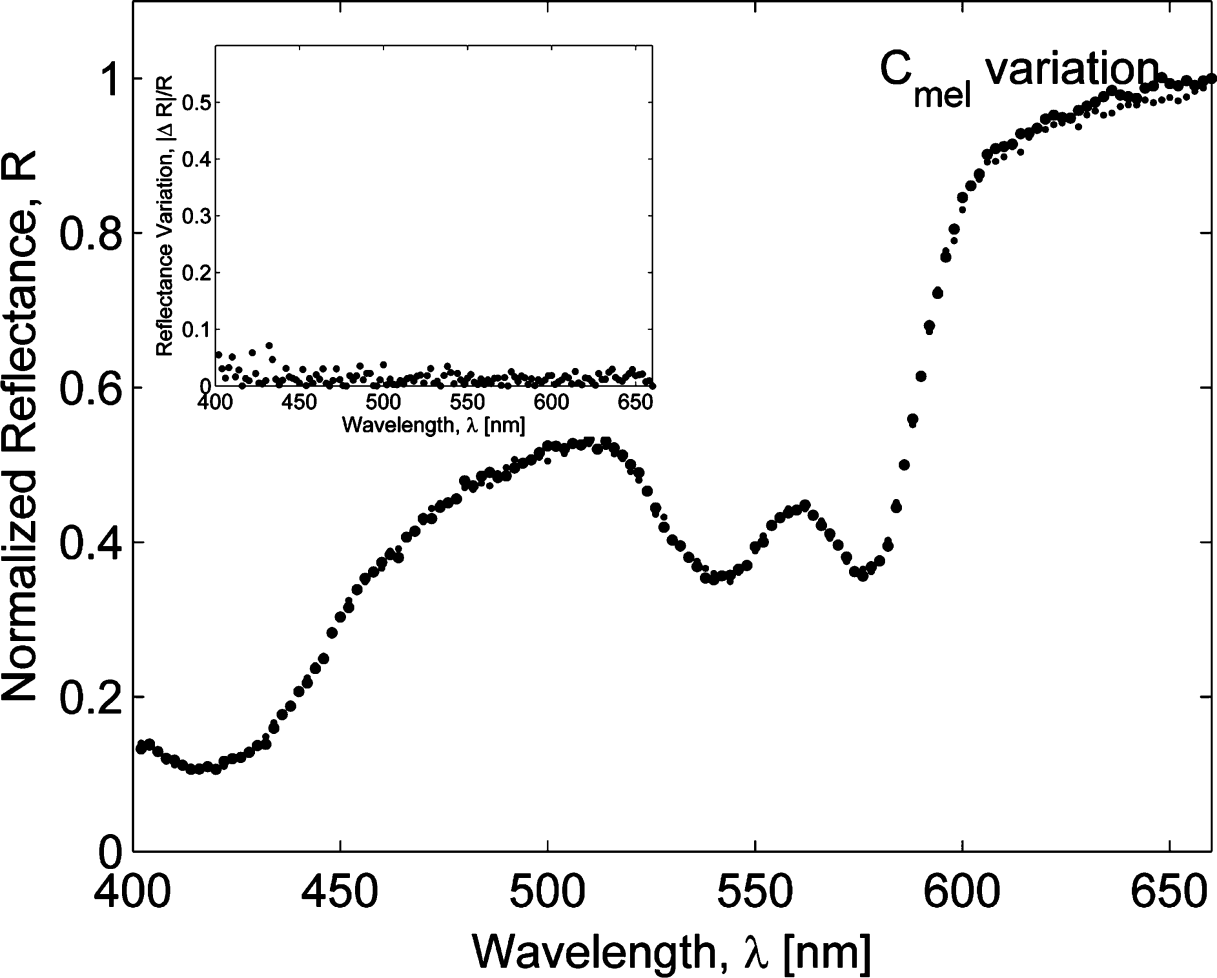
Monte Carlo simulations of palpebral conjunctiva reflectance spectra for different values of melanin concentration *C*_mel_ in the eyelid epidermis. Reflectance spectrum is not sensitive to *C*_mel_.

To assess the effect of variations in eyelid layers thicknesses and chromophore concentrations on a palpebral conjunctiva reflectance spectrum, the sensitivity analysis was performed by calculating the response of the resultant reflectance over 400–660 nm wavelength range to changes in a specific conjunctiva model parameter. First, variations in conjunctiva reflectance were evaluated for different thicknesses of a particular layer, whereas other parameters being fixed. Our simulations showed (Fig. 6) that for a given set of conjunctiva parameter values, the reflectance was most sensitive to variations in the thicknesses of the upper three layers (conjunctival epithelium, tarsal plate, and orbicularis oculi) resulted in up to 30% relative change in reflectance, whereas deeper layers contributed less than 1% to the relative change in reflectance. Next, our simulations demonstrated that changes in blood volume fraction in tarsal plate and orbicularis oculi yielded 40–50% relative variation in conjunctiva reflectance (Fig. 7) and its spectrum is much less sensitive (<2%) to variations in blood volume fraction in subcuteneous tissue and dermis. The sensitivity of the conjunctiva spectrum to the blood oxygenation parameter *S* was evaluated by running simulations for different *S* values varied from 0.3 to 0.9 (Fig. 8). As oxygenation increases in all tissue layers, Hgb becomes more oxygenated and the reflectance spectrum changes according to the total Hgb absorption. The corresponding changes are observed in the shape of the spectral curve: as the amount of oxygenated blood increases, the double peak at around 550 nm becomes more pronounced and the local peak at 440 nm shifts toward shorter wavelengths. Our simulations also showed that the total reflectance was not sensitive to changes of melanin in the epidermis of an eyelid (Fig. 9). For typical skin layer thicknesses and chromophores concentrations (Table 1), relative reflectance variations were less than 1%, when *C*_mel_ changed between 0.01 and 0.1. Thus, the performed sensitivity analysis indicated that the structural complexity of the conjunctiva model can be partially reduced by neglecting factors which impact on the reflectance curve is relatively small.

### Model calibration and predictive simulations

The extended MC model was calibrated using measured experimental spectra of a human conjunctiva. To achieve this, the model was iteratively run and model parameters were varied to find the best fit to experimental data. For a known experimental spectrum, using the measured Hgb and hematocrit values, the model simulated spectra were calculated for various tarsal plate layer thicknesses (1–1.5 mm), blood oxygenation parameter *S* (0.2–0.9), and tarsal layer blood volume fraction (0.1–0.9). The criterion for the best fitted simulated spectrum was to minimize the SSR Σ_*λ*_[*R*_*S*_(*λ*) − *R*_*m*_(*λ*)]^2^, where *R*_*S*_(*λ*) and *R*_*m*_(*λ*) are the simulated and measured reflectance values at the wavelength of *λ*. Thus, the best fit parameters were determined and the mismatch was minimized. After calibration, the MC model was used to simulate reflectance spectra for various Hgb levels in the range from 400 to 660 nm, which corresponds to Hgb level variations in a person whose conjunctiva spectrum was used for calibration.

**Figure 10.**
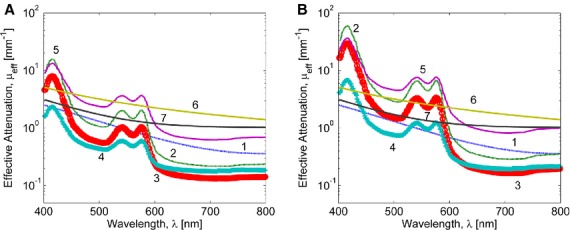
The effective coefficient of attenuation for different eyelid tissue layers calculated according to equation 1: conjunctival epithelium (1), tarsal plate (2), orbicularis oculi (3), subcutaneous fat (4), dermis (5), living epidermis (6), and stratum corneum (7). (A) *S* = 0.6, Hgb = 8.1 g/dL, φ_Hb_ = 0.4; (B) *S *=**0.9, Hgb = 16.4, φ_Hb_ = 0.4.

Light attenuation in deep biological tissues depends on the effective attenuation coefficient defined as 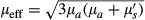, where 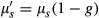. To evaluate light attenuation of different conjunctiva layers, *μ*_eff_ was calculated for different wavelengths using the calibrated input parameters and equations 1–5,1–7. Attenuation of different layers is shown in Figure 10 for Hgb values 8.1 and 16.4 g/dL. As the Hgb concentration increases, the relative attenuation of blood containing layers also increases. Thus, for tarsal plate and orbicularis oculi, *μ*_eff_ increases 1.5 times at *λ *= 800 nm and 3.8 times at *λ *= 415 nm, as Hgb increase from 8.1 to 16.4 g/dL.

**Figure 11.**
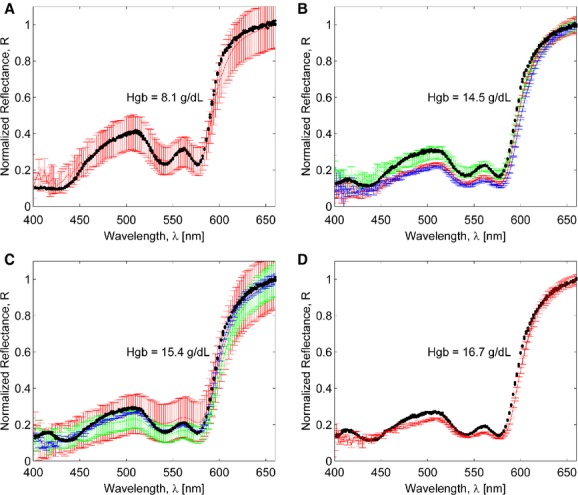
Monte Carlo simulation results for reflectance spectra obtained with the seven‐layered model of a palpebral conjunctiva and comparison with experimental data for different levels of hemoglobin: 8.1 g/dL (A), 14.5 g/dL (B), 15.4 g/dL (C), and 16.7 g/dL (D). The results of model simulations are shown by circles and experimental spectra are shown by lines. Error bars show the standard deviation of experimentally measured spectra of an individual patient. Different colors correspond to different patients having the same hemoglobin level.

Figure 11A–D demonstrates the capability of the model to make predictions of the conjunctiva reflectance for a given set of tissue parameters and a particular Hgb level determined in single‐spectrum calibration. Once the conjunctiva parameters were set (Hgb = 8.1 g/dL, *h*_1_ = 20 *μ*m, *h*_2_ = 100 *μ*m, *h*_3_ = 2 mm, *h*_4_ = 1 mm, *h*_5_ = 300 *μ*m, *h*_6_ = 200 *μ*m), *h*_7_ = 7 *μ*m, 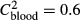, and 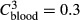), the conjunctiva reflectance spectra were calculated for various Hgb levels up to Hgb = 16.7 g/dL and compared with experimental data (Fig. 1B). Here, the mean experimental values are shown by lines and standard deviations are shown by error bars, based on three individual conjunctiva spectra, measured 30 sec apart. Variations in errorbars (Fig. 1A vs. Fig. 1D) of experimental recordings can be attributed to the fact that averaging was performed over a small number of spectra collected from conjunctivas of both eyes of a patient. One can expect that patient's left and right eyes' conjunctivas vary in tissue structure, Hgb concentrations as well as blood content. The orientation of the light emitter and detector with respect to a conjunctiva surface can also slightly affect the measurements. Meanwhile, the model correctly predicts both qualitative and quantitative behavior of the measured reflectance. The correlation coefficients between the modeled and measured spectra were found to be in the range from 0.992 to 0.996. The simulated and experimental curves agreed within the experimental uncertainty. The SSR values were found to be 0.19, 0.3, 0.18, and 0.17 for (Hgb = 8.1, 14.5, 15.4, and 16.7 g/dL) where each SSR value was calculated with respect to a spectrum averaged over the inter‐ and intrapatient spectra over the wavelengths from 400 to 660 nm.

Figure 11B, C also provide the degree of the intra‐ and interpatient spectral variations for the same Hgb values, which were found to be over 10–20% for the wavelengths below 580 nm. Because individual spectra were taken 30 sec apart, the higher intrapatient spectral variations in this experiment are most likely due to small shifts in sampling locations from one measurement to the next rather than a temporal change in the conjunctiva characteristics within this timeframe. Interpatient variations also points out the importance of collecting multiple spectra from multiple palpebral conjunctiva locations of different individuals to perform a more careful uncertainty analysis. These will be accounted for in future studies.

**Figure 12.**
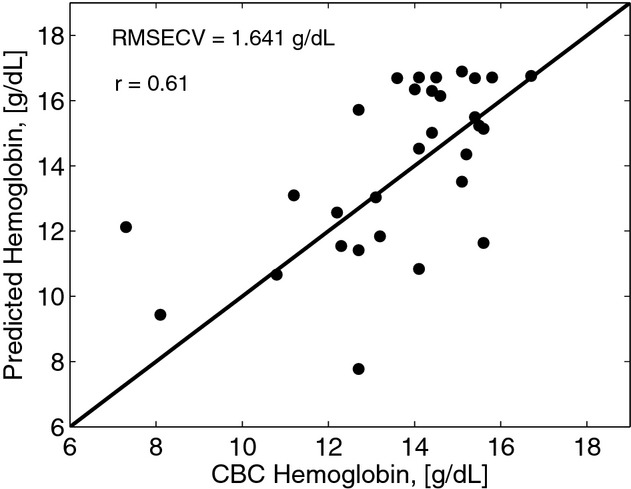
Prediction plot of Monte Carlo (MC)‐based model versus invasive complete blood count (CBC) derived hemoglobin concentration. The correlation coefficient is 0.61 and RMSECV = 1.64 g/dL. RMSECV, root mean squared error of cross‐validation.

**Figure 13.**
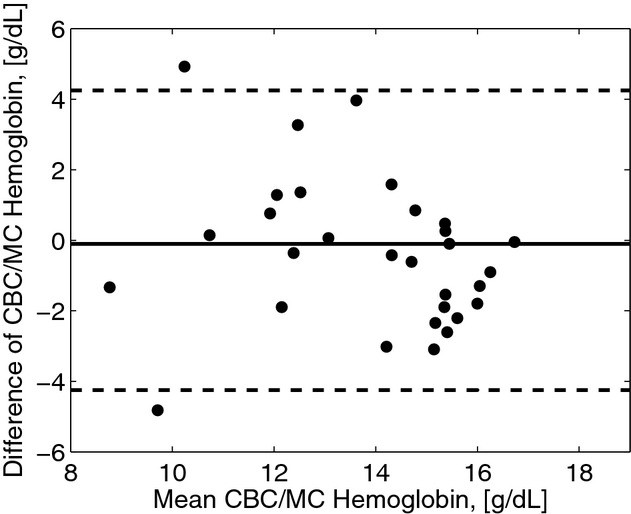
Bland–Altman plot of the model predicted hemoglobin versus the complete blood count (CBC) derived hemoglobin concentration. Solid line is at the mean difference of CBC/Model predicted hemoglobin (−0.1 g/dL) and dashed lines are drawn at ±2 SD (±4.39 g/dL) to represent limits of agreement.

To find the degree of correlation between the predicted and CBC measured Hgb concentrations, we followed the procedure mentioned in the previous subsection and shown in Figure 5. Model calibration was performed by using leave‐one‐out cross‐validation with 29 spectra used as a training set to predict a Hgb concentration corresponding to a single spectrum. For each spectrum of a specific patient, tissue parameters were determined and Hgb and oxygenation levels were varied to simulate multiple spectral curves. Hgb was then extracted by minimizing SSR value for each measured spectrum. This was repeated 30 times such that each spectrum was used once as the validation dataset. After that, the extracted Hgb values and Hgb measured in vitro were cross correlated. The Pearson correlation coefficient was found to be 0.61. The root mean squared error of cross‐validation (RMSECV) was 1.67 g/dL (Fig. 12). For the seven patients whose CBC test showed they were clinically anemic, our method predicted six patients to be clinically anemic yielding the sensitivity of 86%. The results of the model‐based algorithm are also presented as Bland–Altman plot in Figure 13.

From the model simulations, the oxygenation parameter was found to be equal to 0.89, implying that tarsal plate averaged blood oxygenation level was 89%. Although it was not possible to measure the blood saturation level directly in the eyelid tissue, it is worthwhile estimating it. Pulse oximetry measurements provided oxygen saturation of arterial blood (SaO_2_) to vary from 94 to 100% with the mean value of 98.3%. The saturation of venous blood (SvO_2_) varies from 72% to 75% (Sheinberg et al. 1992). Assuming that in the tissue layer oxygenated and deoxygenated blood present in equal parts, the weighted average of the total oxygen saturation varies from 85% to 88% which estimates the value of *S* within 2–7% accuracy.

## Discussion

The goal of this study was to introduce a new method of noninvasive assessment of blood Hgb by combining reflectance spectroscopy of human conjunctiva and stochastic model of light propagation through the conjunctiva tissue. The method has been used to evaluate Hgb levels and anemia conditions and the calculated anemia sensitivity was found to be 86%. We should notice that the obtained anemia sensitivity measure was calculated using one negative result, which was due to the relatively small number of anemic patients (seven) and the overall recruited patient population (30). Larger population study will be needed to improve anemia sensitivity measure.

Due to the presence of multiple parameters characterizing the tissue and high computational cost of the MC simulations, the difficulty in using present MC simulation was in adjusting the simulated reflectance spectra to the measured ones. The total computational cost of finding solution of the inverse problem for one spectrum was ∼24 h using 3.2 GHz processor computer. This corresponds to 30 parameter variation simulations over 200 wavelengths using 10^5^–10^6^ photons for a single‐reflectance spectrum. Although we did not notice that results were strongly affected by the type of the iteration procedure, a more detailed analysis is needed to optimize the choice of the iteration algorithm in the future. Meanwhile, although it is essential to minimize the SSR over the entire spectrum to accurately fit the reflectance curves, focusing on specific characteristic wavelengths such as local extremum or isosbestic points can facilitate predictive simulations by decreasing the number of simulated wavelengths. However, a detailed comparative analysis of how the reduced spectrum affects Hgb calculations would be the goal of future studies.

Variations in conjunctiva parameters from person to person and spatial and wavelength dependencies of optical properties are not well described in the literature. From a few anecdotal human studies, 30–70% conjunctiva thickness variations can be estimated, however, more data should be collected to provide a reliable statistics. Meanwhile, from our simulations, the variation in layers' thicknesses reached 30–100%. Additional studies of human eyelid tissue properties are required to specify the proper distribution of absorption and scattering values. Conjunctiva spectral measurements accompanied by CBC tests of a specific individual over a longer timeframe whose Hgb varies in time will permit to narrow down model parameter uncertainty. These can be achieved by performing measurements during surgery or postsurgery recovery when blood properties can change over time (Luz and Rodrigues 2004). Further parameter optimization can be probably achieved using neural network algorithms although there may be unexpected errors due to local minima (Zhang et al., 2005a).

The computational cost can be reduced by using GPU‐based parallel computing which can provide acceleration from 340 to 1000 times (Alerstam et al. 2008); Doronin and Meglinski 2011). Another potential reduction in the computational costs could be achieved by using adequate simplification of the model based on the sensitivity analysis performed in this study. As we have shown, conjunctiva spectra are mostly affected by the parameters of conjunctiva epithelium, tarsal plate, and orbicularis oculi. This means that potentially, the optical model of the low eyelid tissue can be simplified by considering only the aforementioned three layers, which would be enough to simulate the reflectance spectrum of the palpebral conjunctiva without loss of accuracy. As we have shown, the multilayered planar tissue structure provides very good spectral approximation results. However, further extension of the model would be the use of a higher order spatial approximation of the layer boundaries, such as the one used in (Meglinski and Matcher 2002).Notice that the use of a simpler model such as Kubelka–Munk theory‐based model (KM) (Kubelka 1948) describing radiation transfer in a diffuse scattering medium, is inappropriate in our case. The KM is formulated as a two‐flux model which considers only forward and backward fluxes propagating in an infinite slab of a certain thickness. High‐order approximations of KM has been also reported (Meador and Weaver 1979); Jacques and Prahl 1987); Yang et al. 2004), however, their usefulness is limited because all these models are generally restricted to a simple slab geometries, diffuse irradiance and isotropic scattering, which is atypical for biological tissue optics problems. The relative simplicity and speed of KM models are achieved at the expense of accuracy, which requires a more detailed analysis of the structure and optical properties of different tissue layers. Thus, in our case, the use of a more detailed and complex model based on the inverse MC method and not having the aforementioned limitations, is more appropriate.

## Conclusions

Hgb and blood oxygenation levels are frequently used as indicators of human conditions and different blood diseases such as anemia and hypoxemia. Complementing visual inspection of a patient's conjunctiva with a tool for spectroscopical analysis of the palpebral conjunctiva can potentially provide a reliable noninvasive method to diagnose these conditions. In this study, we have extended our previous studies of conjunctiva reflectance spectroscopy by coupling it with a new modeling approach to predict spectra of patients' conjunctiva.

We have described a method which uses a combination of reflectance spectrometry and stochastic simulation of photon movement in eyelid tissue to allow assessment of a human blood Hgb level. A new seven‐layer tissue model based on histological and optical coherence tomography studies was developed to account for biological details of the human eyelid structure. Different variations in optical parameters were considered to assess the sensitivity of the model. In particular, we showed that the conjunctiva reflectance is sensitive to variations in the thickness of the conjunctiva epithelium, tarsal plate, and orbicularis oculi as well as to variations in blood volume fractions of the tarsal plate and orbicularis oculi.

The experimental conjunctival spectra of adults including anemic patients were used to calibrate the model and run predictive simulations. We showed that the simulated spectra represented a good approximation to the experimentally measured spectra of a human palpebral conjunctiva over the range of different Hgb levels from 8.1 to 16.7 g/dL. The combined predictive simulations and measured spectra of 30 patients permitted us to determine Hgb levels and compare them with Hgb measured in vitro using CBC test. The results of the study demonstrate that combination of measurements using a portable spectroscopic device and a fast computer‐based model analysis software can potentially result in development of an effective novel diagnostic tool for planning the treatment of patients.

## Conflict of Interest

None declared.
